# Noninvasive Assessment of Right Ventricle Function and Pulmonary Artery Pressure Using Transthoracic Echocardiography in Women With Pre-Eclampsia: An Exploratory Study

**DOI:** 10.7759/cureus.13419

**Published:** 2021-02-18

**Authors:** Ahmed F. Zaky, Michael Froelich, Brad Meers, Adam B Sturdivant, Ryan Densmore, Akila Subramaniam, Tekuila Carter, Alan N Tita, Sadis Matalon, Tamas Jilling

**Affiliations:** 1 Anesthesiology, University of Alabama at Birmingham, Birmingham, USA; 2 Anesthesiology and Perioperative Medicine, University of Alabama at Birmingham School of Medicine, Birmingham, USA; 3 Anesthesiology and Perioperative Medicine, University of Alabama at Birmingham, Birmingham, USA; 4 Anesthesiology, University of Iowa, Iowa City, USA; 5 Obstetrics and Gynecology, University of Alabama at Birmingham, Birmingham, USA; 6 Pediatrics, University of Alabama at Birmingham, Birmingham, USA

**Keywords:** pre-eclampsia, echo cardiogram, multifactorial pulmonary hypertension

## Abstract

Background and objective

Pre-eclampsia (PEC) is associated with the release of anti-angiogenic factors that are incriminated in raising systemic and pulmonary vascular resistance (PVR). Compared to the left heart and systemic circulation, much less attention has been paid to the right heart and pulmonary circulation in patients with PEC. We used transthoracic echocardiography (TTE) to estimate pulmonary artery (PA) pressure and right ventricular (RV) function in women with PEC.

Materials and methods

We conducted a case-control study at a tertiary care academic center. Ten early PEC (<34-week gestation) and nine late PEC (≥34-week gestation) patients with 11 early and 10 late gestational age-matched controls were enrolled. Two-dimensional TTE was performed on all patients. The estimated mean PA pressure (eMPAP) was calculated based on PA acceleration time (PAAT). PVR was estimated from eMPAP and RV cardiac output (RV CO). RV myocardial performance index (RV MPI), tricuspid annular plane systolic excursion (TAPSE), tissue tricuspid annular displacement (TTAD), and lateral tricuspid annular tissue peak systolic velocity (S’) were measured.

Results

Compared to early controls, in early PEC, the eMPAP and estimated PVR (ePVR) were elevated, PAAT was reduced, RV MPI was increased, TTAD was reduced, and TAPSE and TV S’ were unchanged. Compared to late controls, in late PEC, the eMPAP and ePVR were elevated, PAAT was reduced, and RV MPI was increased, while TAPSE, TTAD, and TV S’ were unchanged.

Conclusions

In a sample of women with PEC, early PEC was found to be associated with increased eMPAP and ePVR and subclinical decrement of RV function as assessed by TTE. TTE may be a useful noninvasive screening tool for early detection of pulmonary hypertension and RV dysfunction in PEC. An adequately powered longitudinal study is needed to determine the implications of these findings on long-term outcomes.

## Introduction

Pre-eclampsia (PEC) is a leading cause of maternal and perinatal morbidity and mortality worldwide, affecting 1-7% of all pregnancies [1]. The most recent statement by the American College of Obstetricians and Gynecologists (ACOG) has defined PEC as a condition characterized by a systolic blood pressure of ≥140 mmHg or diastolic blood pressure of ≥90 mmHg when measured on two occasions at least four hours apart, and proteinuria, or, in the absence of proteinuria, any signs of thrombocytopenia, renal insufficiency, impaired liver function, pulmonary edema, or cerebral or visual symptoms, all starting after 20 weeks of gestation [2]. The incidence of PEC has been increasing steadily in the US in the last 30 years [3], and about 50-60,000 PEC-related deaths per year have been recorded worldwide [4].

PEC is a vascular disease characterized by the placental production of anti-angiogenic factors such as the soluble form of fms-like tyrosine kinase (sFLT) and soluble form of endoglin (s-ENG). These anti-angiogenic factors by the placenta interfere with adequate placentation, promote a high systemic vascular resistance and placental ischemia or hypoxia, leading to maternal and fetal endothelial dysfunction [5,6]. Recent evidence indicates that an anti-angiogenic milieu, dominated by high plasma levels of the short form of fms-like tyrosine kinase 1 (sFLT-1), similar to what is observed in PEC, is associated with pulmonary hypertension in animals models and in humans [7].

Left ventricular (LV) function and systemic circulation has been studied extensively, by using echocardiography in PEC [8]. Much less attention has been paid to right ventricular (RV) function and to the pulmonary circulation. Given the similarities between the anti-angiogenic milieu in PEC and pulmonary hypertension, we postulate that there may be more significant involvement of the right heart and pulmonary circulation in PEC than previously thought. 

In this study, we utilized echocardiography to explore the changes in RV function and pulmonary circulation in women with early (<34-week gestation) and late PEC (≥34-week gestation). We hypothesize that PEC is associated with echocardiographically estimated RV dysfunction and pulmonary hypertension compared to matched controls.

## Materials and methods

Study design

A cross-sectional study was carried out at the University of Alabama at Birmingham from June 2016 to December 2016 after obtaining approval from the institution's Institutional Review Board (IRB-170119002); written informed consent was obtained from all subjects.

Study population

The inclusion criteria were as follows: adult patients with singleton pregnancy meeting the definition of PEC according to the revised ACOG (2013) criteria [2]. Early PEC was defined as PEC diagnosed at or before 34 weeks of gestation, and late PEC was defined as PEC diagnosed ≥34 weeks of gestation. The exclusion criteria were as follows: multiple gestations, patients with a history of heart failure or ejection fraction (EF) of <40%, valvular heart disease, chronic hypertension, pulmonary artery (PA) stenosis, congenital heart disease, those who were in eclampsia, known diagnosis of pulmonary hypertension due to any cause, RV EF of <45%, and patients who declined to enroll. None of the patients had chronic kidney disease. Patients were enrolled sequentially as identified by the obstetric anesthesiology fellow at the time of daily patient board check-out. A total of 46 patients with singleton pregnancies were enrolled into four groups: early and late PEC with early and late gestational age-matched normotensive pregnant controls. Blood pressure at the time of enrollment was measured manually according to the guidelines of the National High Blood Pressure Education Program Working Group on High Blood Pressure in Pregnancy. All of the measurements were performed in the left arm, in the sitting position, with the arm placed at the level of the heart. Patients underwent physical exams to rule out signs of severe PEC in the form of visual disturbances, headaches, or right upper quadrant abdominal pain. 

Echocardiography

Transthoracic echocardiography (TTE) was performed on all participants upon the diagnosis of PEC and admission to the ward before delivery. All TTE exams were performed by a board-certified single provider (AZ) with a 10-year experience in echocardiography and ultrasonography, using the same device (SPARQ, Philips Vingmed Ultrasound, Horten, Norway) and transducer (S5-1, MHz phased-array). A second board-certified observer (BJM), who was blinded to the patients’ group designation and who had not attended the initial examination, analyzed the echocardiographic exams post hoc. The values entered by the initial examiner were concealed before the examination values were entered by the second observer. Electrocardiography was recorded continuously during echocardiographic studies. Two-dimensional, M-mode, and tissue Doppler TTE imaging were performed according to American Society of Echocardiography guidelines [9].

A conventional, focused, and modified apical four-chamber view (mA4CW), parasternal long axis (PLAX), parasternal short axis (PSAX), left parasternal RV outflow, and the left parasternal RV inflow views were obtained. RV basal diameter (RV-Bd) was measured from mA4CW, focusing on RV at end-diastole. The right atrium (RA) long and short axis diameters (RAd-lax/RAd-sax) and right atrial area (RAA) were measured from mA4CW at end-systole. The RV outflow tract (RVOT) diameter was measured from the left PSAX at end-diastole. RV free wall thickness (RV-FWT) was measured from left PLAX at end-diastole using M-mode. Pulsed-wave Doppler was performed at the mitral, tricuspid, pulmonary, and aortic valves. RV systolic function was evaluated using the fractional area of change (FAC), tricuspid annular plane systolic excursion (TAPSE), and tissue Doppler imaging (TDI) of the peak systolic velocity of the lateral tricuspid annulus. TAPSE was measured using M-mode from the apical four-chamber view. RV diastolic function was estimated using early and late diastolic waves across the tricuspid valve and across the tricuspid annulus. RV myocardial performance index (RV MPI), a representation of the global systolic and diastolic function of the RV, was calculated as described before by using pulsed-wave Doppler velocity and the formula RV MPI = [RV isovolumic contraction time (IVCT)] + RV isovolumic relaxation time (IVRT)/RV ejection time (ET) [10]. RV ET was measured from the pulsed-wave Doppler across the RVOT in the left parasternal RV outflow view. RV MPI reference range was <0.4. RV cardiac output (RV CO) and stroke volume were calculated in accordance with the available guidelines [9].

Apical four-chamber views were specifically optimized to visualize the RV to obtain echocardiographic cine loops by recording three consecutive heart cycles with a frame rate of >60 frames/second. Offline analyses were performed using dedicated software (Qlab 10.3, Philips Healthcare, Eindhoven, Netherlands). To assess tissue-tracking tricuspid annular displacement (TTAD), three user-defined points were selected in the RV-focused view as follows: at the point of insertion of the lateral (TTAD L), and septal (TTAD S) leaflets, and at the midpoint (TTAD MP) of the tricuspid valve to the tricuspid annulus and the RV apex. The software automatically tracked the TTAD and calculated the TTAD at the RV free wall, the TTAD at the interventricular septal wall, and the TTAD at the midpoint of the TV annulus, as well as the percentage displacement of the midpoint (TTAD MP %). In some patients (n=1 in early control, n=2 in early PEC, n=3 in late control, and n=2 in late PEC), the image quality was inadequate to reliably assess TTAD, and in the case of one patient, there was no agreement between the two observers, resulting in the patient's exclusion from the analysis. The apical four-chamber and the PSAX views were used to measure LV EF by the area-length technique [9]. LV endocardial fractional shortening measured by M-mode, FAC, and lateral mitral annular peak tissue velocity measured by TDI were all measured from the parasternal long axis, PSAX, and apical four-chamber views, respectively, according to the available guidelines [9].

Estimation of PA Pressure by Echocardiography

We initially attempted to estimate PA systolic pressure by using the Bernoulli equation (PAP = 4 x TRvel2) from the tricuspid regurgitation jet velocity, in case a tricuspid regurgitant jet was discerned [11]. Since we only had three patients (two patients in early PEC and one patient in late control) with a reliably detectable tricuspid regurgitation jet, these data are not reported.

Since the tricuspid regurgitant jet was not discerned in most cases, the mean pulmonary artery pressure (MPAP) was estimated from the pulmonary artery acceleration time (PAAT) measured across the pulmonary valve using pulsed Doppler waveform by using a logarithmic equation according to the formula validated by Kitabatake et al. [12] and Yared et al. [13], as follows:

eMPAP = 10-0.0068*(PAAT) + 2.1

The PAAT-based analysis was also analyzed by categorical assessment using a cutoff value of PAAT = 100 ms. According to this formula, it was shown that in non-pregnant individuals, PAAT of <100 ms predicts pulmonary arterial hypertension (PAH) and increased pulmonary vascular resistance (PVR) with a sensitivity of 84% and specificity of 90% [14].

PVR was estimated from eMPAP and RV CO using the following formula [15]:

‘Estimated PVR (ePVR) = eMPAP/RV CO’

Estimation of Left Atrial Pressure by Echocardiography

In order to discern whether the elevated MPAP is due to left heart disease versus an increase in ePVR, we utilized a noninvasive method to estimate the filling pressure for the LV, which is based on measuring the E and A waves of the mitral valve (MV) flow velocity and by measuring the velocity of the mitral annulus (e’). Mitral E/A of >2 and E/e’ of >14 show a good correlation with elevated LV filling pressures [16]. 

Statistical analyses

Statistical analyses were performed using the Prism software (GraphPad Software, La Jolla, CA), unless noted otherwise. The primary variables for assessing the differences were eMPAP and PAAT. Since the estimated PA pressure was directly calculated from PAAT, we considered these as a single primary variable. All other variables reported are secondary variables. Continuous variables were presented as mean ±standard deviation. The normality test was calculated by four different methods automatically by Prism: Anderson-Darling (A2*) (AD), D'Agostino-Pearson omnibus (K2) (DP), Shapiro-Wilk (W) (SW), and Kolmogorov-Smirnov (distance) (KS). PAAT, RV MPI, maternal age, gestational age at admission, diastolic BP, heart rate, RV FAC, RV cardiac index (COi), and TAPSE passed the normality tests by all four methods; estimated PA pressure, RV stroke volume index (SVi), systolic BP passed three tests; BMI, ePVR, TTAD S, and TTAD MP % passed two tests; MV E/e’, TTAD L, and TTAD MP only passed DP; MV E/A only passed KS; mean BP and gestational age at delivery did not pass any of the tests. For variables that were not normally distributed. we used the Kruskal-Wallis test with Dunn’s post-hoc analysis. Variables that tested at least three normality tests were considered to be continuous variables, and a two-way analysis of variance (ANOVA) was used with Bonferroni’s post-hoc analysis. Categorical variables were analyzed by using Fisher’s exact test for 2 x 4 tables initially (VassarStats free online calculator developed by Richard Lowry, Ph.D.; http://vassarstats.net/fisher2x4.html), followed by pairwise Fisher’s exact test with Bonferroni’s correction. A p-value of <0.05, after applying the correction for multiple comparisons, was considered to be statistically significant. Inter-observer reliability was determined by using Bland-Altman methodology and expressed as bias (mean difference) and 95% limits of agreement (2 X SD mean difference). The correlation between eMPAP and mean arterial pressure (MAP) was analyzed using linear regression analysis. Since this was an exploratory study with the main purpose to determine whether a larger study was warranted, the sample sizes were not based on power calculation.

## Results

A total of 46 women were enrolled in the study. Six patients were excluded due to the following reasons: delivery at another institution (two), withdrawn consent (two), technical difficulties with echo (one), and non-reassuring fetal heart tones (one), leaving 40 patients for final analysis. The 40 women studied were classified into four groups as follows: early PEC (n=10), matched early controls (n=11), late PEC (n=9), and matched late controls (n=10). The demographic and clinical characteristics of the study groups are presented in Table 1. The mean maternal age and the incidence of smoking were not different across the four groups. The incidence of diabetes in the late PEC group was significantly higher (p=0.011) than in the late control group. BMI was not significantly different between the groups, but all groups had high BMI, and obese patients constituted >50% of subjects in all groups. The mean gestational age in the early groups was significantly different from the late groups, as expected, and there was no difference between the mean gestational age of control and PEC cohorts within the early and late groups.

Systolic arterial pressure was significantly higher in the early and late PEC groups as compared to the corresponding control groups (Table 1). The difference in diastolic arterial pressure was significant only between the early PEC and control groups (Table 1). Heart rates were not significantly different across groups (Table 1).

**Table 1:** Maternal clinical and demographic characteristics SD: standard deviation

Characteristic	Early control (n=11)	Early PEC (n=10)	Late control (n=10)	Late PEC (n=9)
Maternal age (years), mean ±SD	28.4 ±4.9	29.7 ±7.4	27.38 ±4.8	29.2 ±5.8
Gestational age at admission (weeks), mean ±SD	28.8 ±3.3	29.55 ±3.0	36.7 ±1.9 (p=0.0002 vs. early controls)	34.52 ±1.3 (p=0.010 vs. early PEC)
Gestational age at delivery (weeks), mean ±SD	28.8 ±3.3	33.5 ±3.3	37.8 ±1.7 (p=0.002 vs. early controls)	36.7 ±1.1 (p=0.1 vs. early PEC)
Diabetes, n (%)	3 (27)	1 (10)	0 (0)	5 (56) (p=0.02 vs. late controls)
BMI, mean ±SD	38.2 ±18.8	33.7 ±12.0	40.0 ±11.8	34.6 ±8.3
Smoker, n (%)	3 (27)	0 (0)	1 (10)	1 (11)
Systolic arterial pressure (mmHg), mean ±SD	116.3 ±10.6	142.6 ±14.5 (p=0.0008 vs. early controls)	135.8 ±16.4	157.0 ±16.5 (p=0.017 vs. late controls)
Diastolic arterial pressure (mmHg), mean ±SD	72.5 ±11.0	86.5 ±10.0 (p=0.002 vs. early controls)	74.9 ±12.5	81.3 ±9.2
Mean arterial pressure (mmHg), mean ±SD	87.1 ±9.8	105.2 ±10.4 (p=0.009 vs. early controls)	85.7 ±32.6	106.6 ±6.0
Heart rate (BPM), mean ±SD	81.6 ±8.6	93.5 ±16.2	89.0 ±11.5	84.4 ±12.6

**Figure 1:**
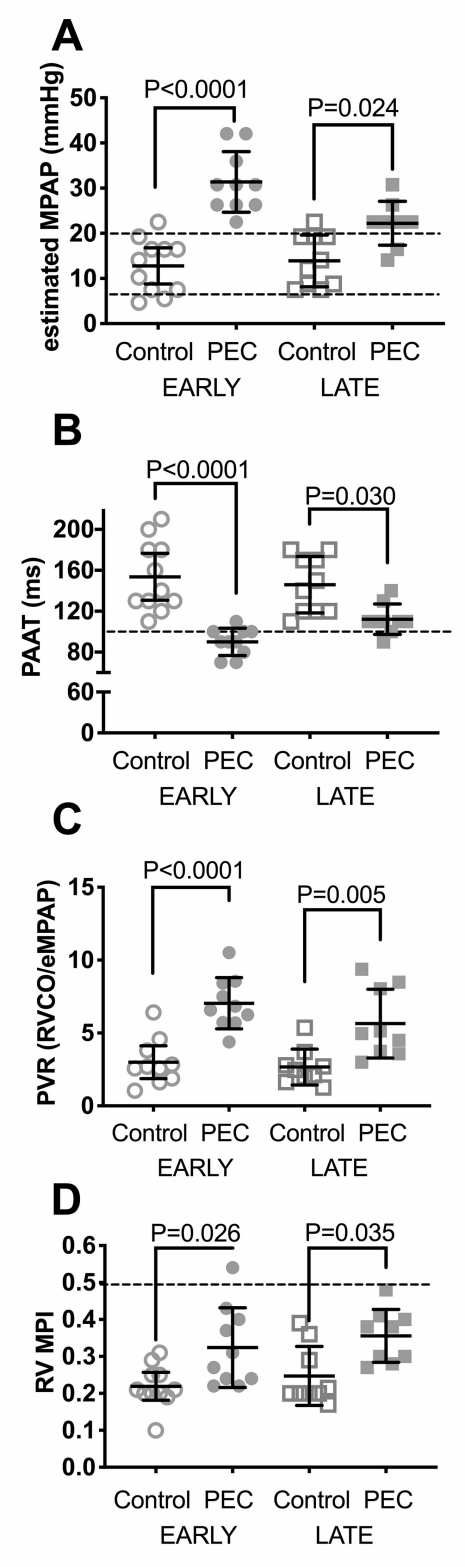
Elevated estimated pulmonary artery pressure and pulmonary vascular resistance in pre-eclamptic women and controls Flow characteristics in the RVOT were examined using TTE as described in Materials and Methods. Data shown are scatter plots of individual measurements overlaid with Mean ±SD, 95% CI of (A) eMPAP. The reference range is indicated by dashed lines. (B) RVOT Doppler waveform PAAT; inversely correlated with PA pressure and pulmonary vascular resistance. A 100-ms cutoff (reference value) is indicated with a dashed line. PAAT of <100 ms detects PAH with a sensitivity of 0.84 and specificity of 0.9. (C) PVR estimated as PVR = (eMPAP)/RV CO. (D) RV MPI. eMPAP, ePVR, and RV MPI increased and PAAT decreased in early PEC as compared to early and in late PEC vs. late controls Statistics by ANOVA with Bonferroni’s post-hoc analysis PVR: pulmonary vascular resistance: eMPAP: estimated mean pulmonary arterial pressure; RVOT: right ventricular outflow tract; PAAT: pulmonary artery acceleration time; PAH: pulmonary arterial hypertension; RV: right ventricle; MPI: myocardial performance index; PA: pulmonary artery; PEC: pre-eclampsia; ANOVA: analysis of variance; RV CO: right ventricular cardiac output

The eMPAP was within the normal reference range (8-20 mmHg) in the early control group (12.8 ±6.0 mmHg, 95% CI: 8.8-16.8), whereas eMPAP in the early PEC group was above the normal reference range (31.4 ±6.7 mmHg, 95% CI: 26.6-36.2), and it was significantly higher than the early control group (12.8 ±6.0 mmHg, 95% CI: 9.8-18.0; p<0.0001) (Figure 1A). Similarly, eMPAP was within the normal reference range in the late control group (13.9 ±5.7 mmHg, 95% CI: 9.8-18.0). The estimated MPAP in the late PEC group was slightly above the normal range (22.2 ±4.9 mmHg, 95% CI: 18.5-26.0), and it was significantly different from the late control group (p=0.024, 95% CI: 18.5-26.0) (Figure 1A). PAAT was lower in early PEC and in late PEC women compared to respective controls (Figure 1B). ePVR was significantly higher in early PEC and in late PEC compared with respective controls (Figure 1C). RV MPI was significantly increased in early PEC (0.32 ±0.11 mmHg, 95% CI: 0.25-0.40) and late PEC (0.36 ±0.07 mmHg, 95% CI: 0.30-0.41) compared to respective controls (early control: 0.22 ±0.06 mmHg, 95% CI: 0.18-0.26) and (late control: 0.25 ±0.08 mmHg, 95% CI: 0.19-0.31) (Figure 1D).

At a PAAT cutoff of 100 ms, there were significantly more patients with early PEC that had a PAAT of <100 ms compared with early controls (Table 2). There was no significant difference in the number of patients with PAAT of <100 ms between late PEC and late controls (Table 2). 

**Table 2:** Distribution of observations based on accepted diagnostic criteria of pulmonary hypertension (at a PAAT of <100 ms) Data were analyzed using Fisher’s exact test, followed by pairwise analysis with Bonferroni’s correction PAAT: pulmonary artery acceleration time; sPAP: systolic pulmonary artery pressure; MPAP: mean pulmonary arterial pressure; PEC: pre-eclampsia

Overall (p=0.00037)	Early control (n=11), n (%)	Early PEC (n=10), n (%)	Late control (n=10), n (%)	Late PEC (n=9), n (%)
PAAT of <100 ms (diagnostic of MPAP >25 mmHg and sPAP >38 mmHg)	0 (0%)	6 (60%) (p=0.008 vs. early controls)	0 (0%)	1 (11%)

RV FAC, indexed RV stroke volume, indexed RV CO, MV E/A, MV E/e’, TAPSE, and TV S’ were not significantly different across any of the groups (Table 3).

**Table 3:** Parameters of right ventricular function Statistics not labeled with * were analyzed by ANOVA with Bonferroni’s post-hoc analysis, and the ones labeled with * were analyzed by Kruskal-Wallis with Dunn’s post-hoc analysis MV: mitral valve; RV: right ventricle; TTAD: tissue tricuspid annular displacement; L: lateral; S: septal; MP: midpoint; MP %: midpoint percentage; SVi: stroke volume indexed to body surface area; COPi: cardiac output indexed to body surface area; TAPSE: tricuspid annular systolic excursion; FAC: fractional area of change; E/A: the ratio of early to late peak diastolic velocity across the mitral valve; E/e’: the ratio of transmitral early diastolic peak velocity to early mitral annular peak diastolic velocity; PEC: pre-eclampsia; ANOVA: analysis of variance

Parameters	Statistics	Early control (n=11)	Early PEC (n=10)	Late control (n=10)	Late PEC (n=9)
MV E/A	P=0.12*	1.46 ±0.33	1.25 ±0.16	1.54 ±0.61	1.22 ±0.29
MV E/e’	P=0.24*	7.70 ±2.14	8.52 ±3.08	7.75 ±2.54	6.53 ±2.84
RV FAC (%)	P=0.30	49.0 ±6.1	41.8 ±10.5	46.0 ±6.1	44.8 ±10.2
RV SVi (mL/m^2^)	P=0.69	23.6 ±7.8	26.8 ±10.2	26.0 ±6.3	28.0 ±7.2
RV COi (L/min/m^2^)	P=0.33	4.1 ±1.2	4.6 ±0.8	5.2 ±1.5	4.4 ±1.6
TAPSE (cm)	P=0.74	2.77 ±0.4	2.71 ±0.1	3.00 ±0.5	2.79 ±0.6
TV S’ (cm/s)	P=0.13	13.2 ±3.9	14.8 ±5.5	11.4 ±3.3	14.8 ±3.1
TTAD (n)		n=10	n=8	n=7	n=7
TTAD L (mm)	P=0.11*	17.3 ±6.4	11.8 ±1.9	19.0 ±5.4	18.0 ±3.5
TTAD MP (mm)	P=0.004*	18.1 ±6.0	8.5 ±4.0 (p=0.008 vs. early control)	16.43 ±5.7	18.0 ±4.7
TTAD S (mm)	P=0.005*	18.7 ±6.2	9.7 ±3.3 (p=0.005 vs. early control)	13.9 ±3.4	17.9 ±5.9
TTAD MP %	P=0.007*	18.0 ±5.6	10.0 ±3.5 (p=0.013 vs. early control)	16.4 ±4.4	17.9 ±4.3

**Figure 2:**
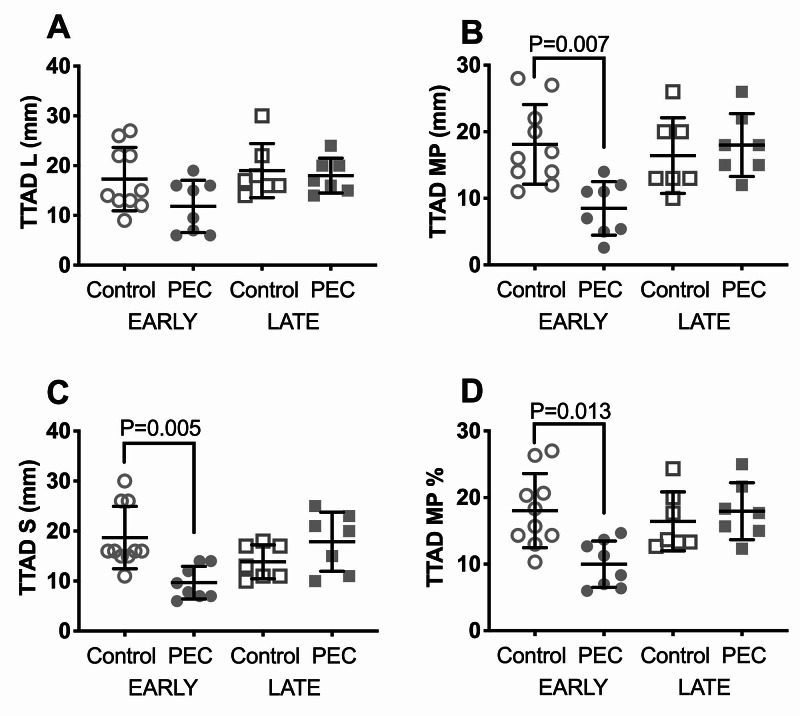
Tissue tricuspid annular displacement (TTAD) in pre-eclamptic women and controls TTAD was assessed at three user-defined points in the RV-focused view as follows: at the point of insertion of the lateral (TTAD L), and septal (TTAD S) leaflets, and at the midpoint (TTAD MP) of the tricuspid valve to the tricuspid annulus and the RV apex. Data shown are individual data points and mean ±SD for each group for TTAD L (A), TTAD MP (B), TTAD S (C), and TTAD MP % (D). In TTAD MP, TTAD S, and TTAD MP %, early PEC was significantly decreased as compared to early controls. Statistics by Kruskal-Wallis; exact p-values are shown PEC: pre-eclampsia; RV: right ventricle; SD: standard deviation

TTAD lateral was not significantly different across groups, but TTAD midpoint, TTAD septal, and TTAD midpoint % were all significantly decreased in early PEC vs. early controls (Table 3) (Figure 2).

**Figure 3:**
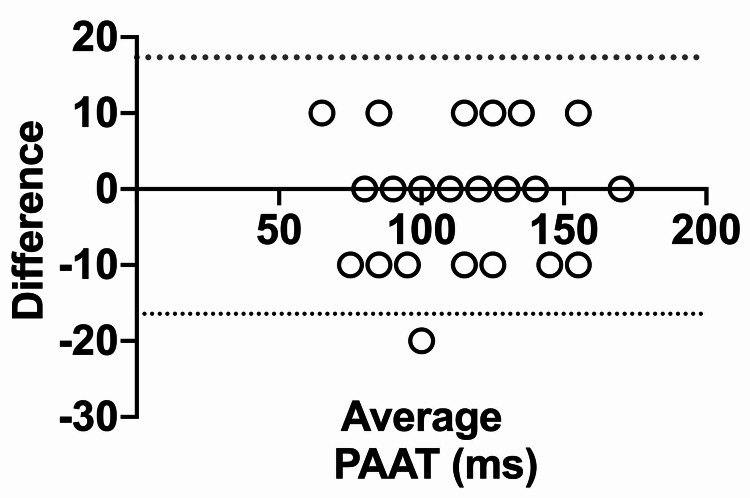
Bland-Altman plot to illustrate inter-observer differences in assessing pulmonary artery acceleration time The plot was generated by analyzing all measurements of PAAT performed by the first and second observers. Dashed lines indicate 95% limits of agreement PAAT: pulmonary artery acceleration time

The Bland-Altman assessment of PAAT between both observers showed a bias close to 0 (0.455) and the SD of bias was 8.61, revealing that there was no systematic bias between the two observers (Figure 3).

**Figure 4:**
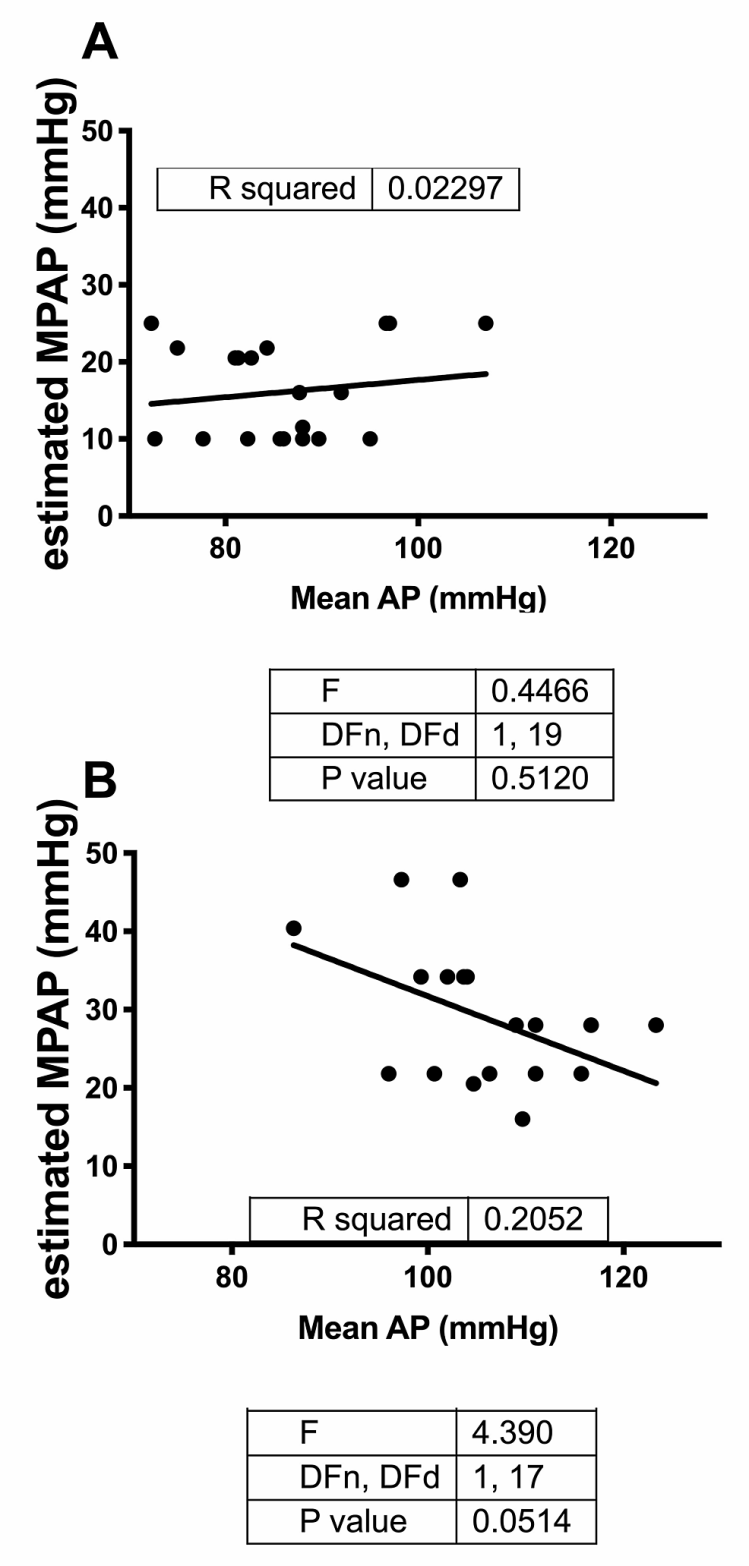
Correlation between mean arterial pressure (MAP) and estimated mean pulmonary artery pressure (eMPAP) MAP and eMPAP were determined as described in Materials and Methods and then plotted as shown. Data shown are as follows: (A) correlation of eMPAP and MAP in all control subjects, and (B) correlation of eMPAP and MAP in all PEC subjects. Linear regression is depicted in each graph by a solid line. The slopes were not significantly different from 0 in either plot by linear regression MAP: mean arterial pressure; eMPAP: estimated mean pulmonary arterial pressure; PEC: pre-eclampsia

The data shown in Figure 1, Figure 2, and Table 3 are by the first observer. Performing the analysis with the data obtained by the second observer resulted in statistical conclusions identical to those obtained by the first observer (data not shown). Patients with a history of heart failure or those with EF of <40% were excluded from the study and none of the originally enrolled subjects had an EF of <55%. In all of the enrolled patients, LV EF measured by the area-length methodology, LV endocardial fractional shortening, FAC, and lateral mitral annular peak S’ wave velocity were within normal limits and there were no statistically significant differences in these parameters of LV function across the groups (data not shown). Additionally, we assessed whether there were signs of elevated left atrial pressure based on recently validated criteria of E/A of >2 or E/e’ of >14 [16]. None of our patients had an E/A of >2 or E/e’ of >14, which ruled out elevated left atrial pressure. Additionally, we plotted the correlation between systemic mean arterial pressure (MAP) and eMPAP. There was no positive correlation between systemic MAP and eMPAP, indicating that PAP was not simply “tracking” elevation of systemic pressure (Figure 4).

## Discussion

In this exploratory study, we demonstrated echocardiographic evidence that suggests a subclinical increase in eMPAP and ePVR, and a modest decrement in RV function in patients with an established diagnosis of early and late PEC compared with age- and gestationally-matched pregnant controls. PAAT at a cutoff value of 100 ms differentiated patients with early PEC compared with controls, substantiating the likelihood of increased PA pressure. Likewise, validated equations that estimate MPAP based on PAAT revealed an increase in eMPAP in PEC compared with controls. ePVR was significantly elevated in both early and late PEC compared to controls. We detected significantly increased RV MPI and reduced midpoint TTAD in early PEC. Other parameters of RV function, such as RV FAC, indexed RV stroke volume, indexed RV CO, tricuspid annular systolic excursion, and TV peak annular tissue velocity were unchanged. In the absence of clinical or echocardiographic evidence of valvular, or systolic, or diastolic LV dysfunction, we speculate that the increased eMPAP, decreased PAAT, and increased ePVR were due to a pulmonary vascular phenomenon rather than a left heart disease etiology. Our study highlights the potential value of echocardiography as a bedside noninvasive tool in the assessment of cardiac structure and function in PEC patients.

Noninvasive assessment of the pulmonary circulation and RV function and structure in PEC is an evolving area of investigation. We are aware of only two studies assessing PA flow dynamics noninvasively in PEC, and both of those studies corroborate our findings in general, although by using a slightly different methodology (without distinguishing between early- and late-onset PEC and without excluding patients with severe PEC) [17,18]. Significantly, although the gold standard for diagnosing PAH is right heart catheterization, it is invasive and increases the risk of maternal and fetal radiation exposure, and is associated with a greater risk of tachyarrhythmias, infection, pulmonary infarction, and PA rupture. Echocardiographic estimation of PAP provides an invaluable noninvasive and safe screening tool for increased PA pressure that warrants further follow-up and investigation. Similarly, echocardiography is a more feasible, bedside option of measuring RV function and structure compared to the gold standard, cardiac MRI [19].

A recent meta-analysis of 21 published studies has indicated that the estimation of MPAP using PAAT shows a very good correlation with values obtained using catheterization, that a cutoff value of PAAT of <100 ms has 84% sensitivity and 90% specificity in the diagnosis of pulmonary hypertension, and that PAAT can be reliably measured in >90% of patients [14]. In our study, we used both the method of using the PAAT of <100 ms cutoff value and also the estimation of MPAP to test whether there was evidence for increased PAP in PEC.

In our early PEC cohort, we found the estimated MPAP to be above the maximum of the normal range. Both Çağlar et al. [17] and Vaught et al. [18] have found significantly increased estimated PA pressures in pre-eclamptic patients, but their reported values were below or at the limit of maximum normal pressure (20 mmHg). The difference could be methodological since they used TV regurgitant jet velocity for their estimate, and we based our estimate on PAAT. Alternatively, the difference may lie in the patient cohort analyzed as our findings suggest a greater effect in early PEC, and Çağlar et al. [17] and Vaught et al. [18] did not analyze early PEC separately.

To the best of our knowledge, our study is the first one to assess TTAD in pre-eclamptic patients. By representing the tricuspid annulus at three points, TTAD may be more representative of the entire longitudinal motion of the tricuspid annulus compared with TAPSE that only measures the free wall tricuspid annular displacement. TTAD L shows a close correlation with TAPSE, and TTAD MP has been shown to be more predictive of RV dysfunction in patients with PAH compared with TAPSE [20] and to better correlate with cardiac MRI-measured RV EF [21]. As such, there was no difference in TTAD L between controls and PEC, whereas TTAD MP was significantly decreased in early PEC vs. early controls.

Reduced RV function in PEC has been shown in previous studies, albeit with the use of different indices of assessment [17,22]. The study by Melchiorre et al. [22] demonstrated biventricular increased wall thickness, and diastolic dysfunction, as well as reduced LV EF and cardiac output. However, we did not find a change in cardiac output or a biventricular diastolic dysfunction. These differences may be related to the study population. Although it was not an exclusion criterion determined apriori, our study sample did not include women with the diagnosis of severe PEC, and we excluded patients with low EF, whereas the study by Melchiorre et al. [22] did not use these exclusion criteria. Additionally, the latter study used TDI to assess strain and strain rate, a technique that has some limitations [23]. The study by Vaught et al. [18] was consistent with our finding of increased RV MPI in PEC patients compared with controls, yet it demonstrated biventricular hypertrophy. This difference may be related to the study population. Vaught et al. [18] studied parturients with early PEC and severe findings, and we excluded patients with the diagnosis of severe pre-eclampsia. Collectively, our study aligns with the literature in terms of the presence of subclinical decrement in RV function and structure and increased PA pressure in patients with early PEC. Whether the mildly reduced RV function is a consequence of increased afterload or whether it develops independently due to circulating mediators is yet to be determined. Intriguingly, a recent study found persistent abnormalities in RV function in women who formerly had early-onset PEC for up to three years post-delivery [24].

Preexisting PAH portends increased mortality in pregnancy and the current recommendation for patients with preexisting pulmonary hypertension is to avoid becoming pregnant, due to a 15-40% incidence of mortality among women who become pregnant with preexisting PAH [25]. There is evidence that PEC and PAH share common mechanisms in their etiology [26,27]. Therefore, we argue that screening and monitoring of the right heart and pulmonary circulation can be further evaluated in a large follow-up study where all parturients are screened.

Our study was exploratory in nature and was designed to provide a proof of concept of the importance of screening for the right heart and pulmonary circulation in PEC and the feasibility of accomplishing this noninvasively. The findings of our study need to be validated using a larger cohort, which will also allow for correlating echocardiographic estimates of MPAP and PVR with indices of disease severity and long-term outcomes. The small sample size precluded appropriate adjustment for comorbidities, which should be addressed in subsequent larger studies. Seven of the 19 PEC patients in this study received antihypertensive therapy during the echo exam, which may have altered the echocardiographic findings compared with controls. However, none of the three drugs, labetalol, hydralazine, or nifedipine, are likely to cause an increase in PA pressure as we observed in this study in the PEC group. Instead, all three drugs have been proposed in the past to be used to treat PAH, although they were not shown to be effective.

Given the cross-sectional design of our study, our echo exams were conducted very close to, yet before, the delivery. Hence the findings may represent a mixed pattern of pregnancy and labor rather than a single pattern. This can be remedied by studying serial exams in future studies. While PEC and control patients were matched by gestational age, there may likely be other systematic differences between the groups that could confound the results. Furthermore, due to the lack of 3D capability in our echocardiography instrument at the time of the study, we were unable to measure 3D volumetric LV EF, which is known to be a more accurate echocardiographic modality for the measurement of LV EF [28]. Furthermore, due to the lack of suitable apical LV two-chamber views, we were unable to measure 2D LV EF using the biplane modified Simpson methodology. The area-length method of LV EF estimation is associated with the limitations of geometric assumption of LV shape as well as sensitivity to foreshortening and wall distortions [9]. Yet, none of our patients had diseases that would lead to LV wall motion abnormalities that are known to affect the accuracy of this methodology. Rather, we used the area-length methodology to estimate LV EF as recommended by the American Society for Echocardiography and European Society of Cardiovascular Imaging. A normal LV FAC and the absence of clinical history or symptomatology of heart failure made us infer that the elevated eMPAP was not due to a left heart pathology. Our study highlights the feasibility and the value of TTE as a diverse screening tool in PEC.

It has to be emphasized that echocardiographic indices of PAH are only estimations and they are used to raise the probability of PAH rather than to establish a conclusive PAH diagnosis.

## Conclusions

The noninvasive assessment of PA hemodynamics and RV function by echocardiography may have utility in assessing the cardiovascular status of PEC patients. Our findings suggest that there is an increase in eMPAP and ePVR in PEC, particularly in early-onset cases. There is also evidence of a parallel mild decrement of RV structure and function. The data we obtained is preliminary, and we recommend that an adequately powered longitudinal study be conducted to assess the clinical significance of our findings, their association with maternal and fetal outcomes, and the condition's responsiveness to antihypertensive therapies.
